# Clinical Characteristics, Neuroimaging Markers, and Outcomes in Patients with Cerebral Amyloid Angiopathy: A Prospective Cohort Study

**DOI:** 10.3390/jcm12175591

**Published:** 2023-08-27

**Authors:** Aikaterini Theodorou, Lina Palaiodimou, Georgia Papagiannopoulou, Odysseas Kargiotis, Klearchos Psychogios, Apostolos Safouris, Eleni Bakola, Maria Chondrogianni, Vasiliki Kotsali-Peteinelli, Konstantinos Melanis, Athanasios Tsibonakis, Elissavet Andreadou, Sofia Vasilopoulou, Stefanos Lachanis, Georgios Velonakis, Elias Tzavellas, John S. Tzartos, Konstantinos Voumvourakis, Georgios P. Paraskevas, Georgios Tsivgoulis

**Affiliations:** 1 Second Department of Neurology, “Attikon” University Hospital, School of Medicine, National and Kapodistrian University of Athens, 12462 Athens, Greece; katetheo24@gmail.com (A.T.); lina_palaiodimou@yahoo.gr (L.P.); georgiapap22@hotmail.com (G.P.); elbakola@yahoo.gr (E.B.); mariachondrogianni@hotmail.gr (M.C.); vkotsali@gmail.com (V.K.-P.); melaniskos@gmail.com (K.M.); tsibonakis-thanos@hotmail.com (A.T.); jtzartos@gmail.com (J.S.T.); cvoumvou@outlook.com (K.V.); geoprskvs44@gmail.com (G.P.P.); 2 Stroke Unit, Metropolitan Hospital, 18547 Piraeus, Greece; kargiody@gmail.com (O.K.); apsychoyio@yahoo.gr (K.P.); safouris@yahoo.com (A.S.); 3 First Department of Neurology, “Eginition” Hospital, School of Medicine, National and Kapodistrian University of Athens, 11528 Athens, Greece; elisandread@gmail.com (E.A.); svassilopoulou@gmail.com (S.V.); 4 Iatropolis Magnetic Resonance Diagnostic Centre, 15231 Athens, Greece; steflach61@gmail.com; 5 Second Department of Radiology, “Attikon” University Hospital, School of Medicine, National and Kapodistrian University of Athens, 12462 Athens, Greece; giorvelonakis@gmail.com; 6 First Department of Psychiatry, “Aiginition” Hospital, School of Medicine, National and Kapodistrian University of Athens, 11528 Athens, Greece; etzavell@med.uoa.gr; 7 Department of Neurology, University of Tennessee Health Science Center, Memphis, TN 38163, USA

**Keywords:** cerebral amyloid angiopathy, cSS, TFNEs, microbleeds, cognitive decline, apolipoprotein E

## Abstract

**Background and purpose:** Sporadic cerebral amyloid angiopathy (CAA) is a small vessel disease, resulting from progressive amyloid-β deposition in the media/adventitia of cortical and leptomeningeal arterioles. We sought to assess the prevalence of baseline characteristics, clinical and radiological findings, as well as outcomes among patients with CAA, in the largest study to date conducted in Greece. **Methods:** Sixty-eight patients fulfilling the Boston Criteria v1.5 for probable/possible CAA were enrolled and followed for at least twelve months. Magnetic Resonance Imaging was used to assess specific neuroimaging markers. Data regarding cerebrospinal fluid biomarker profile and Apolipoprotein-E genotype were collected. Multiple logistic regression analyses were performed to identify predictors of clinical phenotypes. Cox-proportional hazard regression models were used to calculate associations with the risk of recurrent intracerebral hemorrhage (ICH). **Results:** Focal neurological deficits (75%), cognitive decline (57%), and transient focal neurological episodes (TFNEs; 21%) were the most common clinical manifestations. Hemorrhagic lesions, including lobar cerebral microbleeds (CMBs; 93%), cortical superficial siderosis (cSS; 48%), and lobar ICH (43%) were the most prevalent neuroimaging findings. cSS was independently associated with the likelihood of TFNEs at presentation (OR: 4.504, 95%CI:1.258–19.088), while multiple (>10) lobar CMBs were independently associated with cognitive decline at presentation (OR:5.418, 95%CI:1.316–28.497). cSS emerged as the only risk factor of recurrent ICH (HR:4.238, 95%CI:1.509–11.900) during a median follow-up of 20 months. **Conclusions:** cSS was independently associated with TFNEs at presentation and ICH recurrence at follow-up, while a higher burden of lobar CMBs with cognitive decline at baseline. These findings highlight the prognostic value of neuroimaging markers, which may influence clinical decision-making.

## 1. Introduction

Sporadic Cerebral amyloid angiopathy (CAA) is a highly prevalent small-vessel disease, resulting from progressive amyloid-β deposition in the media and adventitia of cortical and leptomeningeal arterioles [1]. CAA is a disease of the elderly with a prevalence of 50–60% among demented elderly populations and a prevalence of 85–95% among patients with Alzheimer’s disease [2,3].

The reference standard for the definite diagnosis of CAA remains the histopathological evidence from brain autopsy or biopsy. However, the introduction of the Boston Criteria (v1.0) in the 1990s has allowed the diagnosis of probable or possible CAA in living patients without conducting a brain biopsy [4]. In 2010, cortical superficial siderosis (cSS) was included in the modified Boston Criteria (v1.5), enhancing their sensitivity for predicting CAA-related hemorrhage [5]. More recently, in 2022, the Boston Criteria version 2.0 for sporadic CAA were published, incorporating non-hemorrhagic, white matter lesions as markers of CAA, further enhancing the sensitivity of these criteria without compromising their high specificity [6].

Patients with CAA may present with various clinical manifestations, including cognitive impairment or dementia, transient focal neurological episodes (TFNEs), and focal neurological deficits due to spontaneous lobar intracerebral hemorrhage (ICH). CAA represents a common etiology of nontraumatic lobar ICH, with a prevalence of about 12% among patients with spontaneous ICH [7]. The mortality of CAA-related ICH varies between 10% and 30% and the rates of ICH recurrence are high (≈10%), even in the absence of antithrombotic treatment [8].

Hemorrhagic lesions, including cortical/subcortical microbleeds (CMBs), lobar ICH, convexity subarachnoid hemorrhage (cSAH), and cSS represent the most prevalent neuroimaging markers of CAA [9,10]. Non-hemorrhagic lesions, such as cortical microinfarcts, white matter hyperintensities (WMHs), enlarged perivascular spaces (EPVS) in the centrum semiovale (CSO), and cerebral atrophy are increasingly recognized among patients with CAA [11,12,13].

Limited data exist regarding the Alzheimer’s disease biomarker levels among patients with CAA. There are controversies regarding the levels of Aβ40, Aβ42, and p-tau proteins and their prognostic value; the majority of reported studies have documented relatively low levels of Aβ42 and Aβ40 in the cerebrospinal fluid (CSF) and high levels of total-tau and p-tau [14,15,16]. Genetic factors, such as the Apolipoprotein-E (APOE) genotype, may also play a significant role in the pathophysiology of CAA. For instance, the ε2 allele has been strongly associated with disseminated cSS among patients with symptomatic CAA [17].

In this prospective, single-center cohort study, our primary aim was to assess the prevalence of baseline characteristics, clinical manifestations, and neuroimaging findings among patients with CAA, and to analyze long-term outcomes. Additionally, we aimed to examine the levels of CSF Alzheimer’s biomarkers and the prevalence of various APOE genotypes.

## 2. Materials and Methods

The data that support the findings of the present study are available from the corresponding author upon reasonable request. This study was performed in accordance with the Strengthening the Reporting of Observational Studies in Epidemiology (STROBE) guidelines for reporting observational research [18]. Informed consent was obtained from all participants or their legal representatives before enrollment.

### Participants

The study was conducted at our tertiary stroke center in “ATTIKON” University Hospital, in accordance with the Declaration of Helsinki principles. Institutional review board approval was obtained from the ethics committee of “Attikon” University Hospital (decision number: EBΔ 499). We performed a prospective, single-center, hospital-based cohort study including all consecutive patients with the diagnosis of CAA according to the modified Boston Criteria v1.5 admitted to our department during a five-year period (2018–2022) [5]. The patients presented either at our stroke service or at our outpatient stroke and dementia clinic.

Major exclusion criteria were a history of aneurysmal subarachnoid hemorrhage, a history of ICH located in the brainstem or supratentorial deep grey nuclei (basal ganglia or thalami) and white matter, indicative of hypertension-related small-vessel disease (i.e., lipohyalinosis) and the presence of isolated infratentorial siderosis. Patients without available brain MRI or with missing sequences, including susceptibility-weighted imaging sequences (SWI), gradient echo T2*-weighted sequence (GRE-T2*), 3D fluid-attenuated inversion recovery sequences (FLAIR), and T2-weighted sequence, were excluded. Patients with Cerebral amyloid angiopathy-related inflammation (CAA-ri) and patients with hemorrhagic lesions suggestive of CAA that were attributed to other disorders (e.g., cavernous malformations, malignancies, or radiation-induced cerebral vasculopathy) were also not included in our study. Finally, patients who did not provide consent to participate in the current study were also excluded.

## 3. Materials

Data on (1) demographics (sex and age), (2) cardiovascular risk factors (arterial hypertension, dyslipidemia, diabetes mellitus, atrial fibrillation (AF), current smoking, history of excessive alcohol intake), (3) antiplatelet or anticoagulant treatment at presentation, (4) clinical manifestations and neuroimaging markers at presentation, and (5) CSF biomarker profile and APOE genotype were collected. All patients were prospectively followed for at least 12 months and brain MRI was repeated at the 12-month follow-up visit or in case of new symptoms.

### 3.1. MRI Acquisition

All patients underwent either a 1.5 Tesla (Philips Healthcare, Best, the Netherlands) or, preferably, a 3 Tesla MRI scan (Siemens Magnetom Prisma, Siemens Healthineers, Erlangen, Germany). All participants were examined using a comprehensive protocol, including the GRE-T2* sequence or the SWI sequence, the T2-weighted sequence, the 3D FLAIR sequence, the diffusion-weighted imaging (DWI) sequence, and the post-contrast T1-weighted sequence. Two attending-level radiologists (G.V. and S.L.), who were blinded to clinical characteristics, evaluated all neuroimaging studies.

The following markers were assessed following the Standards for Reporting Vascular Changes on Neuroimaging (STRIVE) criteria: CMBs, cSS, cSAH, and/or EPVS in the CSO using a dichotomized classification (high (≥21 EPVS) or low (≤20 EPVS)) and white matter hyperintensities according to the Fazekas Scale [19,20,21]. In addition, we assessed cortical microinfarcts and cerebellar hemorrhagic lesions.

The presence and distribution of CMBs were evaluated on SWI or GRE-T2* images [21]. cSS was defined as linear residues of chronic blood products in the superficial layers of the cerebral cortex showing a characteristic “gyriform” pattern of low signal on SWI or T2*-GRE images, without corresponding hyperintense signal on T1-weighted or FLAIR images (i.e., without acute subarachnoid hemorrhage). We did not include cSS contiguous with any ICH. The distribution of cSS was classified as focal (restricted to ≤3 sulci) or disseminated (>3 sulci) [5,22]. cSAH was detected in blood-sensitive MRI sequences with characteristic blooming limited to the subarachnoid space of the convexities, not extending into the parenchyma, sylvian fissures, ventricles, or basal cisterns. In some patients with available brain computed tomography, cSAH was seen as hyperintense lesions in the same region.

### 3.2. Cerebrospinal Fluid Biomarkers and APOE Genotype

Lumbar puncture was performed at the L4–L5 interspace according to our department’s state-of-the-art protocol. The CSF was collected in polypropylene tubes, centrifuged, aliquoted, and stored in polypropylene tubes at −80 °C. Samples with more than 500 red blood cells/μL were discarded. CSF Aβ40, Aβ42, tau phosphorylated at threonine 181, and total tau levels were blindly quantified in duplicate using the Lumipulse chemiluminescent immunoassay (Fujirebio, Gent, Belgium). The samples were analyzed in different batches; however, we adhered to strict guidelines and the manufacturer’s instructions.

Peripheral blood leukocytes were collected from 18 patients, who provided additional consent for the extraction of genomic DNA. APOE genotypes were determined using polymerase chain reaction (PCR) and direct sequencing. The genotypes ε3/ε3, ε2/ε3, and ε4/ε3 were recorded, according to the different combinations of codons 112 and 158 of the fourth exon of the APOE gene.

### 3.3. Neuropsychological Assessment

Following a complete physical and neurological examination, a neuropsychological test assessing global cognitive function through the use of Mini-Mental State Examination (MMSE) was performed [23]. Patients with neurological symptoms such as aphasia or patients who were unable to cooperate, were excluded.

### 3.4. Follow-Up Assessments and Outcomes

All patients were followed for at least 12 months and follow-up data were prospectively collected. We recorded information on clinically symptomatic ICH, defined as a symptomatic stroke syndrome associated with neuroimaging evidence of a corresponding ICH (>10 mm in diameter), or radiologically isolated and clinically silent ICH detected in follow-up imaging or death from any cause. Outcome events were assessed using all available clinical and radiological information, blinded to the presence of cSS and CMBs at baseline MRI.

## 4. Statistical Analysis

For dichotomized variables, the chi-square test (sample size > 5) or the Fisher exact test (sample size ≤ 5) was used. The Shapiro–Wilk test was used to analyze the normality of the continuous data. If parameters were normally distributed, they were depicted as mean ± standard deviation; otherwise, they were stated as medians with interquartile ranges (IQR). Continuous variables were tested with the Student’s *t*-test (normally distributed data) or Mann–Whitney U-test (non-normally distributed data).

Univariable and multivariable binary logistic regression models were used to evaluate the associations of different parameters with clinical outcomes before and after adjusting for potential confounders. A cutoff of *p* < 0.1 was used to select variables for inclusion in multivariable analyses that were conducted using a backward stepwise selection procedure. To confirm the robustness of multivariable models, we repeated all multivariable analyses using a forward selection procedure. Associations are presented as odds ratios (ORs) with corresponding 95% confidence intervals (CIs). Statistical significance was achieved if the *p* value was ≤0.05 in multivariable logistic regression analyses.

Moreover, Cox proportional hazard regression models were used to calculate the associations of baseline characteristics, clinical manifestations, and neuroimaging findings with the risk of recurrent ICH. For patients experiencing multiple symptomatic ICH events during follow-up, data were censored after the first symptomatic ICH event. All covariates demonstrating a univariable association with the outcome in Cox regression analysis (*p* < 0.1) were considered for inclusion in the multivariable model. The proportional hazard assumption in unadjusted and adjusted models was tested using graphical checks and Schoenfeld residuals-based tests. All tests of significance were 2-tailed, and the significance level was set at 0.05 for all analyses. All statistical analyses were conducted using the R software version 1.4.1717 (R Foundation for Statistical Computing, Vienna, Austria) [24].

## 5. Results

### 5.1. Participant Characteristics

**Figure 1:**
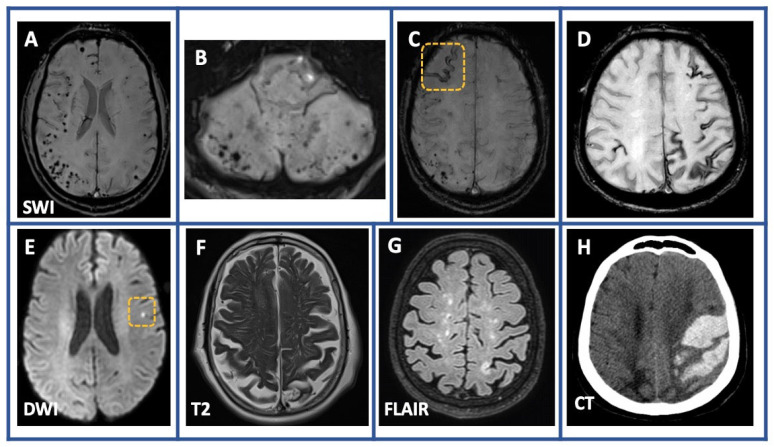
Common neuroimaging findings among patients with Cerebral amyloid angiopathy. Susceptibility-weighted imaging (SWI) sequences showing multiple cerebral (panel **A**) and cerebellar microbleeds (panel **B**) as well as focal (panel **C**) and disseminated (panel **D**) cortical superficial siderosis. Diffusion-weighted imaging (DWI) sequence (panel **E**) demonstrating a cortical microinfarct left parietal. Non-hemorrhagic lesions such as enlarged perivascular spaces in centrum semiovale and multispot white matter hyperintensities pattern are depicted in T2-weighted image (panel **F**) and 3D fluid-attenuated inversion recovery sequences (FLAIR) sequences (panel **G**), respectively. Large lobar intracerebral hemorrhage with characteristic finger-like projections is depicted on the baseline computed tomography of one of our patients (Panel **H**).

**Figure 2:**
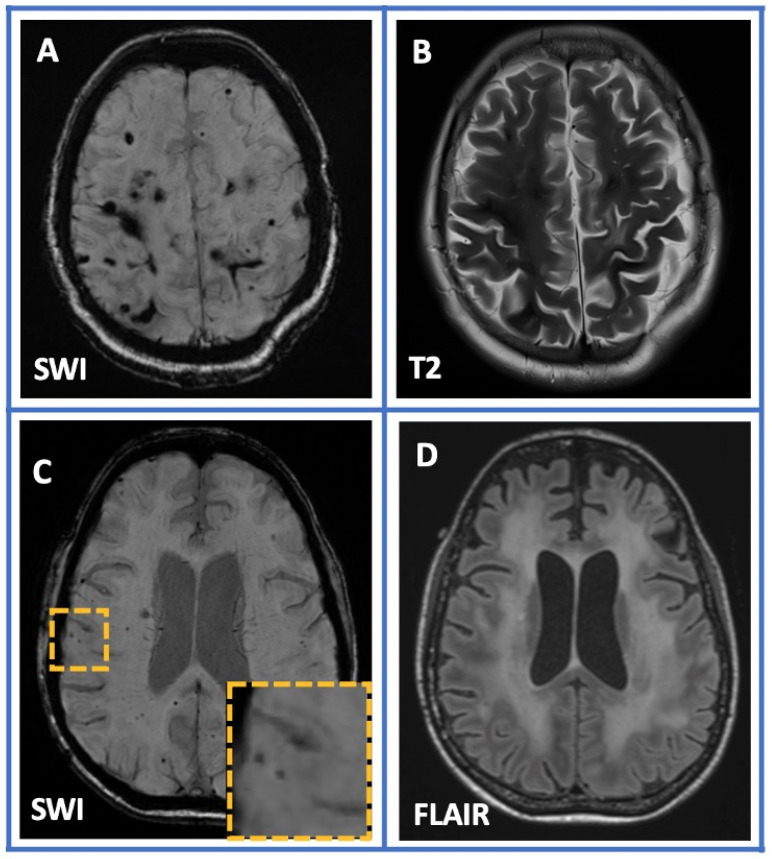
Conditions mimicking CAA. A 56-year-old female patient with multiple cerebral hemorrhagic lesions attributed to multiple cerebral cavernous malformations (panels **A** and **B**). Susceptibility-weighted imaging (SWI) sequence (panel **C**) showing radiation-induced multiple cerebral microbleeds associated with extensive bilateral white matter hyperintense lesions (panel **D**) in a 64-year-old man with known metastatic melanoma.

All patients underwent a brain MRI at baseline (Figure 1). Patients with neuroimaging lesions suggestive of CAA which were attributed to other diseases, including multiple cerebral cavernous malformations, radiation-induced white matter hyperintensities associated with hemorrhagic lesions, cerebral amyloidoma, and CAA-ri, were excluded from our cohort (*n* = 14; Figure 2).

The final cohort consisted of 68 patients fulfilling the revised Boston diagnostic criteria v1.5 for CAA: 53 (78%) with probable CAA and 15 (22%) with possible CAA. Baseline characteristics including demographics, vascular risk factors, clinical features, neuroimaging markers, CSF biomarker levels, and genetic findings of the study participants are summarized in Table 1. The mean age of the study participants was 71 ± 8 years old and 53% (36/68) of the study population were men. There was no autopsy/biopsy-based diagnosis and no history of brain surgery at a younger age among the study patients. Seventy-one percent (48/68) of the participants had a history of arterial hypertension and twelve percent (8/68) had a history of AF. Particular attention was given to patients with arterial hypertension regarding any possible white matter lesions indicative of hypertensive small vessel disease. No specific lesions were found in these patients who fulfilled the modified Boston Criteria v1.5 for CAA. A neuropsychological assessment was performed on 54 patients (4 patients with aphasia, 7 who were unable to cooperate and complete the test, and 3 who denied this assessment were excluded from the neuropsychological examination).

### 5.2. Clinical Features and Neuroimaging Markers at Presentation

The prevalence of focal neurological signs, cognitive impairment, and TFNEs was 75% (51/68), 58% (39/68), and 21% (14/68), respectively; 25% (17/68) of the study patients complained of headaches at initial presentation, 28% (19/68) presented with behavioral changes or psychiatric symptoms, and 16% (11/68) with seizures.

The core neuroimaging findings are summarized in Table 1 (Figure 1). Ninety-three percent of the patients (62/68) had lobar CMBs, and forty-three percent (29/68) presented with lobar ICH. cSS was disseminated in 31% (21/68) of patients and focal in 16% (11/68). cSAH was also present in 13% (9/68) of patients. Cerebellar microbleeds were detected in 39% (26/68), whereas cerebellar cSS was not detected in our cohort. Non-hemorrhagic lesions, including EPVS in CSO and multispot white matter pattern, were detected in 50% (34/68) and 50% (34/68) of participants, respectively. Nineteen percent (13/68) of the patients had cortical microinfarcts as well. With regard to underlying AF, there were no significant (*p* < 0.05) differences in the prevalence of clinical manifestations and neuroimaging findings between patients with and without AF history.

Moreover, a brief comparison between the main findings of this cohort and the results from a recent meta-analysis regarding the prevalence of different CAA clinical and neuroimaging markers is provided in Table 2 ^7^. It is important to note that, in both studies, the most common clinical features were cognitive impairment and TFNEs, while CMBs and cSS were the most prevalent hemorrhagic markers.

### 5.3. Cerebrospinal Fluid Biomarkers and APOE Genotypes

Lumbar puncture was performed on 28 patients, and the remaining 40 patients declined the procedure. Alzheimer’s disease biomarkers analysis is summarized in Table 1. The results demonstrated mean Aβ40 levels of 6697.0 ± 1684.5 pg/mL, decreased levels of Aβ42 (452.4 ± 204.9 pg/mL (cut-off value for CSF Aβ42: 520 pg/mL)), and increased levels of total tau (528.9 ± 347.8 pg/mL (cut-off value for CSF total tau: 360 pg/mL)) and phosphorylated tau protein (67.8 ± 36.9 pg/mL (cut-off value for CSF p-tau: 60 pg/mL)). The ratio Aβ42/Aβ40 was 0.076 ± 0.038 (cut-off value for ratio Aβ42/Aβ40: 0.063).

Genetic findings revealed 11 carriers (61%) of the APOE ε3/ε3 genotype, 4 patients (22%) with the ε2/ε3 genotype, and 3 patients (17%) with the ε4/ε3 genotype.

### 5.4. Predictors of TFNEs and Cognitive Impairment at Presentation

The univariable and multivariable associations of baseline characteristics with the likelihood of initial clinical presentation with TFNEs are presented in Table 3. The following variables were associated with TFNEs on initial univariable analyses using a *p*-value of <0.1 as the threshold for inclusion in multivariable models: age, male sex, arterial hypertension, lobar ICH, lobar CMBs, greater burden of CMBs (>10), cerebellar CMBs, cSAH, cSS, disseminated versus focal cSS, multispot white matter hyperintensities pattern, EPVS in CSO, and cortical microinfarcts. Only the presence of cSS was independently (*p* < 0.05) associated with higher odds of TFNEs as initial presentation in multivariable logistic regression analyses conducted using the backward selection procedure (OR:4.504, 95% CI:1.258–19.088, *p*-value:0.027). We repeated the multivariable analyses using the forward selection procedure and obtained identical results.

Respectively, the univariable and multivariable associations of baseline characteristics with the likelihood of initial clinical presentation with cognitive impairment are presented in Table 4. The following variables were associated with cognitive impairment on initial univariable analyses using a *p*-value of <0.1 as the threshold for inclusion in multivariable models: age, male sex, arterial hypertension, lobar ICH, lobar CMBs, greater burden of CMBs (>10), cerebellar CMBs, cSAH, cSS, disseminated versus focal cSS, multispot white matter hyperintensities pattern, EPVS in CSO, and cortical microinfarcts. Only the greater burden of CMBs was independently (*p* < 0.05) associated with higher odds of cognitive decline as initial presentation in multivariable logistic regression analyses conducted using the backward selection procedure (OR:5.418, 95% CI:1.316–28.497, *p*-value:0.027). We repeated the multivariable analyses using the forward selection procedure and obtained identical results.

### 5.5. Follow-Up Data and Predictors of ICH Recurrence

The median follow-up time was 20 months (range 12–38). At the end of the follow-up, 65 patients were alive, whereas 3 patients died following a major ICH. Among the eight patients with coexistent AF, three patients underwent left atrial appendage occlusion without complications.

During the follow-up, 2 of 68 patients (3%) experienced a symptomatic cerebral infarction, and, in 6 patients (9%), new cortical silent microinfarcts were documented in the follow-up brain MRI. Moreover, 19 of 68 patients (28%) experienced recurrent ICH. Out of these 19 patients, 4 patients experienced multiple recurrent ICH. It is worth mentioning that the vast majority of these patients (87%) had no underlying arterial hypertension, nor had they received antihypertensive medication during the follow-up period. In multivariable Cox regression models, cSS emerged as the only risk factor of recurrent ICH (HR:4.238, 95% CI:1.509–11.900, *p*-value:0.006).

## 6. Discussion

Our prospective single-center study of consecutive CAA patients showed that the most common clinical features were focal neurological signs, cognitive decline, and TFNEs. The most prevalent neuroimaging characteristics were hemorrhagic lesions, including CMBs and ICH with lobar distribution and focal or disseminated cSS. Non-hemorrhagic lesions including EPVS in CSO, multispot white matter pattern hyperintensities, and cortical microinfarcts were frequently recognized. The Alzheimer’s biomarkers profile of our cohort was characterized by decreased levels of Aβ42 and Aβ40 and increased levels of total tau and *p*-tau protein. The presence of cSS and the greater burden of CMBs were associated with the greater likelihood of TFNEs and cognitive decline as initial presentation, respectively. Focal/disseminated cSS was also related to a higher risk of ICH recurrence among CAA patients.

The clinical spectrum of CAA includes various manifestations [9]. Acute focal neurological deficits, accompanied sometimes by headache and/or epileptic seizures, are the consequences of intracerebral lobar hemorrhage [25]. Spontaneous ICH remains the most common presentation among patients with CAA, with a prevalence rate of about 44%, as demonstrated in a recent meta-analysis [9]. This rate is compatible with the ICH prevalence of 43%, as observed in our cohort. Due to the amyloid deposition within the cortical and leptomeningeal vessels, CAA-related ICH is common in lobar locations, with a predominance in temporal and occipital lobes [25].

CAA-related TFNEs, also called amyloid spells, are of brief duration (<30 min) and characterized by recurrent, stereotyped episodes of focal symptoms, with spreading progression mimicking a migratory spread [26]. Patients with TFNEs describe more often positive symptoms, including aura-like spreading paresthesias, limb jerking, and visual paresthesias. Negative symptoms, such as sudden-onset limb weakness or aphasia, which resemble transient ischemic attacks, are less common [27]. CAA-related TFNEs are attributed to either cSAH or more commonly to focal or disseminated cSS, which represents the deposition of chronic blood breakdown products within the supratentorial, subarachnoid space, and along the superficial cortical layers [26]. Moreover, TFNEs and the presence of cSS are strongly associated with a higher risk of future symptomatic spontaneous lobar ICH. A recent study showed increased ICH incidence among patients with motor TFNEs and four times higher ICH incidence among patients who also received antithrombotic agents [28]. Comparing patients with and without cSS, the presence of cSS among CAA patients doubles the risk of any ICH in the future, and in cases with disseminated cSS, the risk is four times higher [29]. These observations are also compatible with our cohort study results, which documented the focal/disseminated cSS as an independent predictor of recurrent ICH.

Amyloid deposition in the cortical and leptomeningeal vessel walls has been shown to be associated with cognitive impairment and dementia, which are considered as another core clinical manifestation among patients with CAA, with an estimated prevalence of CAA among demented elderly populations of about 50–60% [1,30]. Despite the fact that in Alzheimer’s disease brains, CAA histopathology is identified in approximately 80% of patients, the overall cognitive profile of CAA is more similar to that seen in classic vascular cognitive impairment [2,31]. In CAA, processing speed and executive functions are more severely impaired than memory [31,32]. Various neuroimaging markers, including total brain atrophy, the history of lobar ICH, and the presence of cSS or cortical microinfarcts, have been associated with worse cognitive performance and a higher risk of progression to dementia [32,33]. Our findings also indicate that a higher burden of CMBs is strongly associated with a higher risk of cognitive impairment, leading probably to dementia.

The role of CSF biomarkers in the diagnosis and management of patients with CAA remains uncertain. It is worth mentioning that a substantial case number of patients (one of the highest in the literature) underwent lumbar puncture in our cohort, providing critical information about CSF biomarker levels in CAA. Our results documented decreased levels of Aβ42 and Aβ40 and increased levels of total-tau and p-tau proteins, confirming findings from previous cohorts [14,15,16,34]. The utility of these biomarkers is controversial but may be of clinical value when the diagnosis of CAA is in doubt or in order to detect an underlying CAA pathology among healthy controls or Alzheimer’s disease patients. The differentiation between CAA and AD on the basis of standard CSF biomarkers, such as Aβ40, Aβ42, total tau, and phosphorylated tau protein, has been proven to be difficult or even unsuccessful [16,34]. In the current literature, a combined panel with novel CSF biomarkers, such as Aβ38 and Aβ43, has been proposed [16]. This seems to have greater potential to distinguish CAA from AD. In any case, this may potentially help exclude patients with a high load of CAA from various anti-Aβ immunotherapy clinical trials [35,36]. Furthermore, since the recently published Boston v2.0 diagnostic criteria for CAA have only 55% sensitivity in patients without ICH, the CSF biomarkers panel could add more diagnostic accuracy in this subgroup [6].

Despite that CAA is increasingly recognized nowadays due to the greater availability of high-resolution brain MRI images, neurologists should have a comprehensive understanding of this entity and a continuous vigilance to differentiate it from other diseases that could radiologically mimic CAA. A very cautious interpretation of neuroimaging findings is important in order to identify cases of cavernous malformations, malignancies such as central nervous system lymphoma, diffuse axonal injuries, radiation-induced cerebral vasculopathy, genetic disorders including cerebral autosomal dominant arteriopathy with subcortical infarcts and leukoencephalopathy (CADASIL), and COL4A1 gene mutations [37,38,39,40,41,42]. CAA-ri, as a rare subtype of CAA, should be included in the differential diagnosis in cases with coexisting radiological evidence of inflammation and typical CAA findings [43]. In these cases, the early diagnosis and the prompt treatment initiation with immunosuppressive therapies could improve the prognosis and disease evolution [44].

The present study has certain limitations that should be acknowledged. First, the sample size of the study was moderate (N = 68). Second, unwillingness to undergo diagnostic procedures, including lumbar puncture and genetic testing, was the main reason for the limited data regarding the CSF biomarker profile and the APOE genotypes in our cohort study. Third, in none of our patients, the diagnosis relied on histopathological confirmation. It should also be noted that, due to the moderate sample, we did not conduct any subgroup analysis. With respect to genetic markers, previous studies have highlighted possible associations of APOE ε2+ with the pathophysiology and severity of cSS or, alternatively, a dose-dependent association between APOE ɛ4 and sporadic CAA [17,45]. Due to limited data, we were not able to explore/determine associations between different polymorphisms and clinical phenotypes in our cohort. Another potential limitation that needs to be taken into consideration is the selection of patients using the Modified Boston Criteria v1.5 given the fact that data collection started and ended before the publication of the modified Boston Criteria v2.0. Further prospective validation of our results is required in larger multicenter cohorts and registries.

In summary, the present study provides a comprehensive overview of the various clinical manifestations and radiological markers among Greek patients with CAA. Moreover, our findings lend support to the current evidence regarding the associations of cSS with TFNEs at presentation and ICH recurrence during the follow-up period. These results highlight the prognostic value of neuroimaging markers, which may influence clinical decision-making in CAA patients.

## Figures and Tables

**Table 1:** Baseline characteristics of our study population (*n* = 68).

Variable	Overall (*n* = 68)
Age at presentation (years), mean (SD*)	70.9 (8.3)
Sex—male, *n* (%)	36 (52.9)
Diagnosis based on biopsy/autopsy, n (%)	0 (0.0)
Diagnosis based on Boston Criteria v.1.5 Definite—probable—possible, n (%)	0 (0.0)–53 (77.9)–15 (22.1)
History of brain surgery, n (%)	0 (0.0)
3 Tesla brain MRI SWI vs. GRE-T2* sequence	52 (76.5%) 57 (80.9%)
Follow-up (months), median (IQR*)	20 (12–40)
Vascular risk factors	
Arterial hypertension, n (%)	48 (70.6)
Hyperlipidemia, n (%)	38 (55.9)
Diabetes mellitus, n (%)	14 (20.6)
History of atrial fibrillation, n (%) Left atrial appendance occlusion, n (%)	8 (11.8) 3 (37.5)
Antiplatelet use at presentation	18 (26.5)
Anticoagulant use at presentation	6 (8.8)
Treatment with rtPA at presentation	1 (0.01)
Clinical signs at presentation	
Signs of intracerebral hemorrhage, n (%)	29 (42.6)
Focal neurological signs, n (%)	51 (75.0)
TFNEs, n (%)	14 (20.6)
Cognitive impairment, n (%)	39 (57.4)
MMSE—score, median (IQR*)	24 (21–27)
Headache, n (%)	17 (25.0)
Seizures, n (%)	11 (16.2)
Behavioral changes/ psychiatric signs, n (%)	19 (27.9)
Neuroimaging findings at presentation	
Lobar hemorrhage, n (%)	29 (42.6)
Lobar cerebral microbleeds, n (%)	62 (92.5)
Cerebellar microbleeds, n (%)	26 (38.8)
cSAH, n (%)	9 (13.2)
cSS, n (%) Disseminated cSS vs. focal cSS, n (%)	32 (47.8) 21 (30.9)
Cerebellar cSS, n (%)	0 (0.0)
Enlarged perivascular spaces in centrum semiovale, n (%)	34 (50.0)
Multispot white matter hyperintensities pattern, n (%)	34 (50.0)
Cortical microinfarcts, n (%)	13 (19.4)
Gd+ enhancement, n (%)	4 (0.06)
CSF biomarkers and APOE-genotype	
APOE 3/3–2/+–4/+	11 (61.1)–4 (22.2)–3 (16.7)
Total tau	528.9 (347.8)
Phospho–tau	67.8 (36.9)
Amyloid β40	6697.0 (1684.5)
Amyloid β42	452.4 (204.9)
Aβ42/ Aβ40	0.076 (0.038)

SD: standard deviation, IQR: interquartile range.

**Table 2:** Comparison of the results from the present cohort and a recent meta-analysis evaluating clinical and neuroimaging characteristics in CAA patients.

Clinical Phenotypes and Neuroimaging Markers	Present Cohort	Recent Meta-Analysis
Lobar cerebral microbleeds	93%	52%
cSS	48%	49%
ICH	43%	44%
Microinfarcts	19%	30%
High grades of perivascular spaces located in centrum semiovale	50%	56%
White matter hyperintensities	50%	53%
MCI/dementia	57%	50%
TFNEs	21%	48%

**Table 3:** Univariable and multivariable logistic regression analyses depicting the associations of baseline characteristics with the likelihood of initially presenting with TFNEs among patients with Cerebral amyloid angiopathy.

Variable	Univariable Logistic Regression Analysis		Multivariable Logistic Regression Analysis	
	Odds Ratio (95% CI)	*p*-Value *	Odds Ratio (95% CI)	*p*-Value *
Age	1.055 (0.979–1.141)	0.169		
Age (per 10-year increase)	1.634 (0.780–3.579)	0.200		
Male (sex)	0.207 (0.043–0.756)	0.027	0.628 (0.169–2.263)	0.475
Arterial hypertension	1.676 (0.448–8.155)	0.472		
Radiological Findings at Presentation				
ICH	-	0.993		
Lobar CMBs	0.153 (0.018–1.024)	0.053	0.276 (0.024–2.504)	0.256
Number of lobar CMBs (>10)	0.299 (0.069–1.377)	0.106		
Cerebellar CMBs	0.509 (0.126–1.750)	0.304		
cSAH	2.046 (0.385–9.143)	0.361		
cSS	3.750 (1.091–15.229)	0.045	4.504 (1.258–19.088)	0.027
Disseminated vs. focal cSS	2.154 (0.395–16.938)	0.404		
Multispot white matter Hyperintensities pattern	2.604 (0.758–10.540)	0.145		
EPVS in CSO	0.960 (0.288–3.203)	0.946		
Cortical microinfarcts	4.607 (1.21–17.861)	0.024	1.209 (0.244–5.631)	0.808

* cutoff of *p* < 0.1 was used for selection of candidate variables for inclusion in multivariable logistic regression models.

**Table 4:** Univariable and multivariable logistic regression analyses depicting the associations of baseline characteristics with the likelihood of initially presenting with cognitive impairment among patients with Cerebral amyloid angiopathy.

Variable	Univariable Logistic Regression Analysis		Multivariable Logistic Regression Analysis	
	Odds Ratio (95% CI)	*p*-value *	Odds Ratio (95% CI)	*p*-value *
Age	1.024 (0.965–1.090)	0.441		
Age (per 10-year increase)	1.353 (0.732–2.581)	0.341		
Male (sex)	1.569 (0.586–4.284)	0.372		
Arterial hypertension	0.694 (0.223–2.051)	0.515		
Radiological Findings at Presentation				
ICH	2.639 (0.951–7.789)	0.068	2.616 (0.843–8.873)	0.105
Lobar CMBs	2.188 (0.338–17.568)	0.41		
Number of lobar CMBs (>10)	5.333 (1.343–26.967)	0.024	5.418 (1.316–28.497)	0.027
Cerebellar CMBs	1.295 (0.471–3.647)	0.618		
cSAH	1.613 (0.384–8.248)	0.527		
cSS	2.625 (0.954–7.606)	0.067	1.613 (0.384–8.248)	0.527
Disseminated vs. focal cSS	1.250 (0.210–6.602)	0.794		
Multispot white matter Hyperintensities pattern	0.688 (0.247–1.875)	0.466		
EPVS in CSO	0.929 (0.339–2.529)	0.884		
Cortical microinfarcts	0.553 (0.157–1.895)	0.344		

* cutoff of *p* < 0.1 was used for selection of candidate variables for inclusion in multivariable logistic regression models.

## Data Availability

The datasets used and analysed during the current study are included in this article. More detailed datasets are available from the corresponding author on reasonable request.
